# *In vivo* evaluation of safety, biodistribution and pharmacokinetics of laser-synthesized gold nanoparticles

**DOI:** 10.1038/s41598-019-48748-3

**Published:** 2019-09-09

**Authors:** Anne-Laure Bailly, Florian Correard, Anton Popov, Gleb Tselikov, Florence Chaspoul, Romain Appay, Ahmed Al-Kattan, Andrei V. Kabashin, Diane Braguer, Marie-Anne Esteve

**Affiliations:** 1 Aix Marseille Univ, CNRS, INP, Inst Neurophysiopathol, Marseille, France; 2 Aix Marseille Univ, CNRS, INSERM, Institut Paoli-Calmettes, CRCM, Marseille, France; 3 APHM, Hôpital de la Timone, Service Pharmacie, Marseille, France; 4 Aix Marseille Univ, CNRS, LP3, Campus de Luminy, Case 917, 13288 Marseille, France; 5 MEPhI, Institute of Engineering Physics for Biomedicine (PhysBio), Bio-nanophotonics Lab., 115409 Moscow, Russia; 6 Aix Marseille Univ, Avignon Université, CNRS, IRD, IMBE, Marseille, France; 7 APHM, Hôpital de la Timone, Service d’Anatomie Pathologique et de Neuropathologie, Marseille, France

**Keywords:** Nanotechnology in cancer, Methods of toxicology studies

## Abstract

Capable of generating plasmonic and other effects, gold nanostructures can offer a variety of diagnostic and therapy functionalities for biomedical applications, but conventional chemically-synthesized Au nanomaterials cannot always match stringent requirements for toxicity levels and surface conditioning. Laser-synthesized Au nanoparticles (AuNP) present a viable alternative to chemical counterparts and can offer exceptional purity (no trace of contaminants) and unusual surface chemistry making possible direct conjugation with biocompatible polymers (dextran, polyethylene glycol). This work presents the first pharmacokinetics, biodistribution and safety study of laser-ablated dextran-coated AuNP (AuNPd) under intravenous administration in small animal model. Our data show that AuNPd are rapidly eliminated from the blood circulation and accumulated preferentially in liver and spleen, without inducing liver or kidney toxicity, as confirmed by the plasmatic ALAT and ASAT activities, and creatininemia values. Despite certain residual accumulation in tissues, we did not detect any sign of histological damage or inflammation in tissues, while IL-6 level confirmed the absence of any chronic inflammation. The safety of AuNPd was confirmed by healthy behavior of animals and the absence of acute and chronic toxicities in liver, spleen and kidneys. Our results demonstrate that laser-synthesized AuNP are safe for biological systems, which promises their successful biomedical applications.

## Introduction

Gold (Au) is one of very important elements of the periodic table, known by both luxury qualities and unique physical, physico-chemical and chemical characteristics. The reduction of size of gold down to the nanoscale leads to novel astonishing properties such as prominent catalytic^1,2^ and electrocatalytic^3,4^ responses, a variety of plasmonic effects associated with optical excitation of collective free electron oscillations (surface plasmons), including strong resonant extinction^5^, drastic field enhancement^6^, optical manipulations beyond the diffraction limit^7^, ultrasensitive biosensing^8^, etc. On the other hand, gold is highly biocompatible^9,10^, which promises successful biomedical applications of Au nanoparticles (AuNP) profiting from plasmonic or other effects^11^. Oncology looks as one of most promising application areas, as after an appropriate functionalization AuNP can be targeted to the tumor (actively or passively), accumulated in cancer cells and then be used either as contrast agents in cancer imaging or sensitizers of different local therapies under external stimuli. As an example, extremely efficient optical absorption, with typically ~10^5^-fold higher cross section than absorbing dyes, has been used in a number of diagnostic and therapeutic modalities, including light induced hyperthermia-based therapy^12–14^, confocal reflectance microscopy^15^, photoacoustic tomography^16^, optical coherence tomography^17^ imaging modalities. For these optical modalities, Au nanostructures should have their absorption/scattering feature within the tissue transparency window between about 700 and 1000 nm, which can be satisfied by employing designed plasmonic structures (SiO_2_-Au or Si-Au core-shells, Au nanorods). As another prominent example, relatively small (5–20 nm) AuNP can serve as contrast agents in X-ray radiation imaging^18,19^ and as sensitizers of radiation therapies^20–22^.

Such biomedical applications require the choice of minimally toxic Au-based nanomaterials to enable such imaging or therapeutic functionalities. The toxicity of Au nanostructures depends on size and shape (spherical, nanorods, nanostars, etc) characteristics, as well as surface conditioning and chemistry, including the presence of toxic contaminants, surface charge and stabilization coatings^23–26^. Conventional methods for the synthesis of colloidal Au nanostructures (nanoparticles, nanorods, core-shells) are based on colloidal chemistry, including reverse micelles or the reduction of a gold precursor in the presence of capping agents.^13–15,27–30^, but these methods cannot provide optimal parameters for biomedical applications. First, these methods typically cause surface contamination by residual by-products, ligands and reducing agents, which can cause toxicity issues^31–33^. Second, in many cases chemically-synthesized AuNP are positively charged or stabilized by cationic surfactants, which can provoke their undesirable interactions with negatively charged DNAs^34–36^. Third, the stabilization of chemically-synthesized AuNP and advanced structures (nanorods, nanocages, nanostars etc.) typically requires surfactants and protecting ligands that can lead to severe toxicity effects. As an example, the stabilization of nanorods is based on the use of cetyl trimethylammonium bromide (CTAB)^25,26^, which causes unexpectedly high toxicity^24,37^. The biocompatibility of chemically-synthesized Au nanostructures can be much improved by their coating by polymers such as polyethylene glycol (PEG), dextran, polyvinyl-pyrrolidone (PVP), poly (acrylic acid) (PAA), and polyvinyl-alcohol (PVA)^23,24^, but such coating cannot completely protect from the release of toxic agents after a long-term exposure of AuNP-polymer complexes *in vivo*. The evolution of AuNP in biological systems, including efficiency of cellular uptake, biodistribution etc., is finally highly affected by a protein corona (layer of proteins that adsorb on the nanoparticles in biological environments), but the composition of this corona is also strongly affected by initial surface conditioning and coatings^23–25^. In general, spherical nanoparticles with the size around 15–50 nm look less toxic, provided they do not contain toxic contaminants and are properly coated^23–26^.

Laser-ablative synthesis was recently introduced as a “green” physical alternative to conventional chemical pathways, which offers quite different surface conditioning and chemistry, as well as an exceptional level of material purity^38–41^. Such a synthesis profits from natural generation of nanoclusters under ablation of a solid target by laser radiation in liquid ambient^42^. The nanoclusters then coalesce in the liquid to form colloidal nanoparticle solution. Since this method does not depend on the presence of specific chemical products (e.g., gold salts) during the synthesis, it can avoid any contamination of nanoparticle surface, while the use of ultrashort (femtosecond) laser ablation enables to finely control size and structural characteristics of formed AuNP^40,41,43–45^. It is also important that laser-synthetized AuNP exhibit unusual surface chemistry based on the presence of gold oxide states, which conditions O^−^ termination when pH >5^46^. Such a surface chemistry, hardly reproducible with chemical synthesis pathways, makes possible hydrogen bonding interactions of AuNP with a variety of biocompatible molecules containing OH groups, including oligosaccharides^46,47^, biopolymers (polyethylene glycol (PEG), dextrans)^48^, proteins^49^ and oligonucleotides^50^. The one-step conjugation with polymers or other molecules (no intermediate group is required) presents an interesting new option, which could give some advantages for biological applications. In addition, laser-ablated AuNP (bare or polymer-coated) are typically negatively charged, which promises the absence of toxic effects related to DNA damage.

Ultrapure AuNP prepared by laser ablation could be considered as new appealing candidate for biomedical applications, but there are only a few data describing their interaction with biological systems. Studying the interaction of laser-synthesized AuNP with rat pancreatic tumour cells, Goldys *et al*.^51^, reported their non-toxicity and a size-dependent cellular uptake. Barcikowski *et al*. showed that the functionalization of laser-produced AuNP with some peptides^52^ and polymers^53^ can improve cell uptake. We recently studied cytotoxicity and cell uptake of three types of Au nanoparticles: bare (ligand-free) AuNP, dextran-coated AuNP and PEG-coated AuNP^54^. Cell viability profiles demonstrated that AuNP did not induce major inhibition of cell survival until concentrations of 100 µg/mL, indicating a satisfactory low toxic effect, while all type of AuNP demonstrated a good cellular uptake. By examining the interaction of bare, dextran- and PEG-coated AuNP with living cells, for the first time we determined the composition of the protein corona covering the nanoparticles in biological environment. Such a corona appeared after 30 seconds of AuNP exposition and contained a variety of proteins, including apolipoproteins, which are implicated in intracellular transport and crossing of biological barriers, while the presence of immune proteins (complement C3) was lower for polymer-coated AuNP compared to bare ones, suggesting their better transport properties.

This study is conceived as the first attempt to assess the interaction of dextran polymer-coated laser-synthesized AuNP with biological systems *in vivo*. Using intravenous administration of AuNPd in mouse model, we examine toxicity, biodistribution and pharmacokinetics of these nanoparticles. We show that despite certain residual accumulation in organs of reticuloendothelial system, laser-synthesized AuNPd look safe and do not provoke any sign of toxic effects.

## Results

### Synthesis and characterization of dextran-coated Au nanoparticles

**Figure 1:**
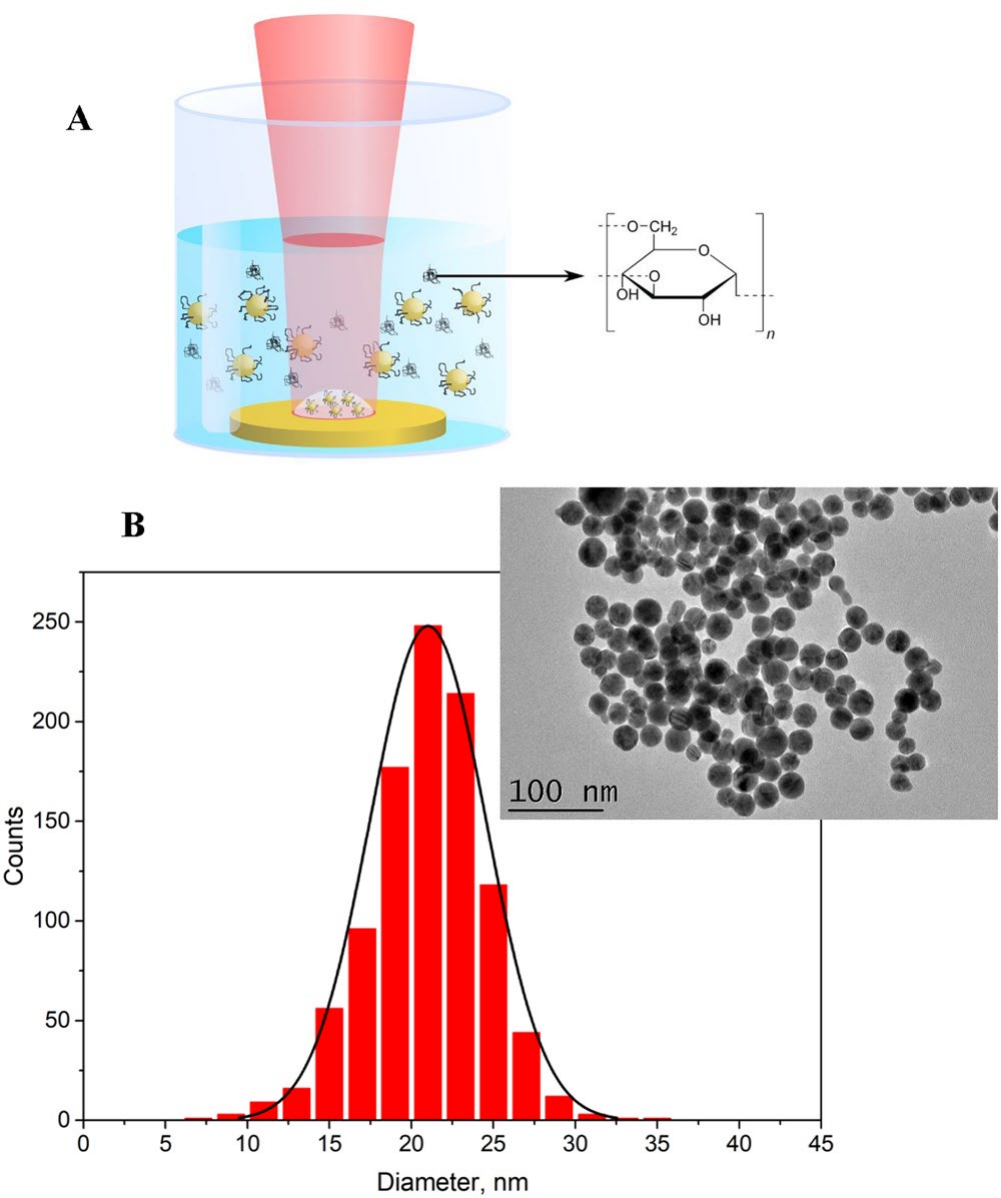
(**A**) Schematics of laser ablation setup. A laser beam is focused on the surface of the Au target, which is placed in the vessel filled with an aqueous solution of dextran. (**B**) Typical transmission electron spectroscopy (TEM) image and corresponding size distribution of AuNP synthesized by pulsed laser ablation in aqueous solution of dextran.

For fabrication of AuNP, we used a technique of fs laser ablation in aqueous solutions, which was developed in our previous studies^46–48^. A gold target was placed on the bottom of a glass vessel filled with an aqueous solution of dextran (1 mg/mL) and ablated by radiation from a femtosecond Yb:KGW laser (see details in Methods section), as shown in Fig. 1A. The ablation process led to the production of colloidal dispersions of AuNP, which was accompanied by a characteristic red coloration of solutions. The formed solutions exhibited a characteristic peak around 520 nm in the extinction spectra, which is associated with the excitation of surface plasmons over gold NP. As we showed in our earlier studies^46–48,54^, Au nanoparticles prepared by the technique of femtosecond laser ablation in aqueous solutions exhibit exceptionally high stability (they are stable for several years while keeping at room temperature) due to electrical repulsion effect resulting from strong negative charging of nanoparticles. In our case, AuNPd were also very stable, as we did not see any sign of agglomeration or precipitation after several months of storage in glass vials. According to transmission electron microscopy (TEM) images and size distribution analysis (Fig. 1B), particles prepared in dextran solution had the mean size of 21 nm and size dispersion less than 10 nm full-width-at-half maximum. As we earlier showed^54^, hydrodynamic diameter of such dextran-coated AuNPd is about 46 nm, which is consistent with the formation of Au nanoparticle – dextran complexes.

### Effects of AuNPd on body weight

**Figure 2:**
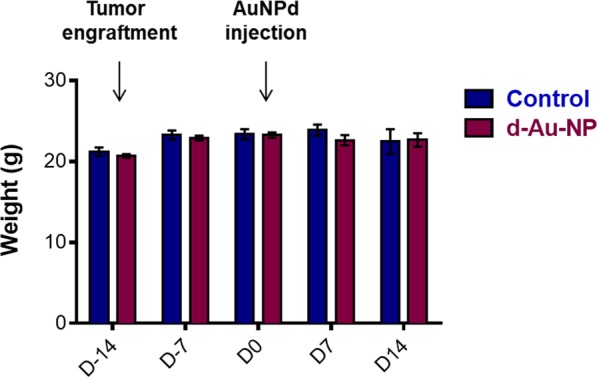
Evolution of the mean body weight of control mice or mice exposed to 1 mg/kg AuNPd. Mice were weighted twice a week throughout the 28 days of the follow-up experiment; data represent the mean ± SEM of 6 mice.

Three groups of six mice were administered with a single intravenous dose of 1 mg/kg of AuNPd and the control group was injected with vehicle alone. To investigate potential toxic effects due to NP, body weight and mice behavior were monitored after AuNPd administration. All animals showed a healthy appearance and normal activity without lethargy or apathy after NP administration. During the first 14 days after subcutaneous tumor grafting, as well as during the 14 days following AuNPd injection, the body weight of the mice injected with NP slightly increased in a pattern similar to the control mice, suggesting a normal mice growth in the absence of any significant toxic effect (Fig. 2). Three mice of each group were placed in metabolic cages 24 h before the sacrifice (which occurs 24 h, 7 days or 14 days after AuNP administration), to monitor their food and water consumption and to collect urines for further determination of gold content. We did not observe any difference in water intake or food consumption between treated and control groups.

### Pharmacokinetic analysis

**Figure 3:**
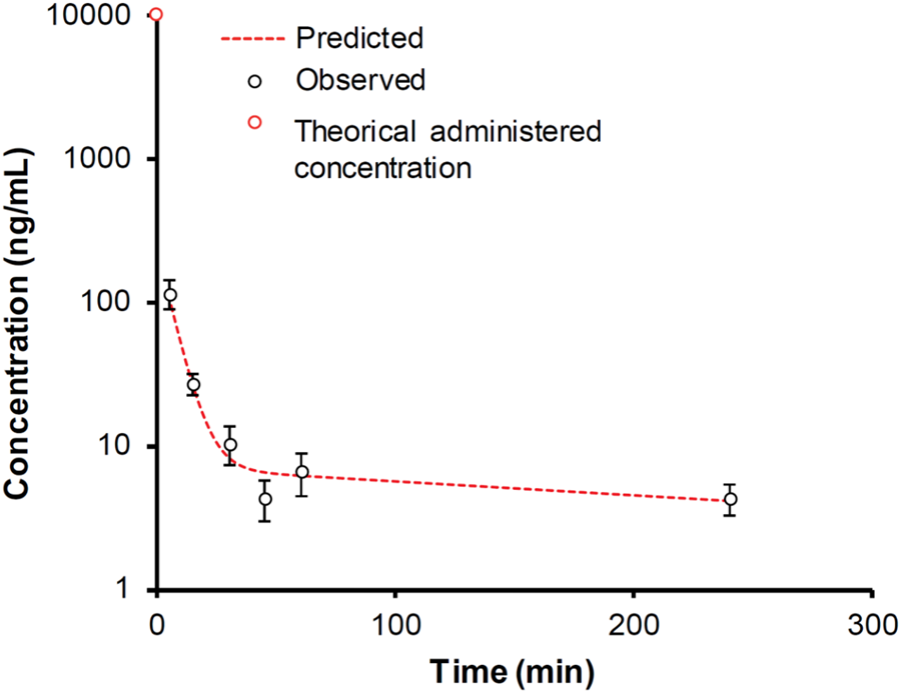
Pharmacokinetic profile of AuNPd (1 mg/kg) at 5, 15, 30, 45, 60 min and 4 h after a single intravenous administration (n = 5, data are mean ± SEM). A point for 0 minute corresponds to theoretical administered concentration (10 000 ng/ml).

To determine pharmacokinetic profile of AuNPd in blood, seven groups of five athymic nude female mice were treated with AuNPd at a unique dose of 1 mg/kg by IV injection. Gold concentration was determined by inductively coupled plasma mass spectrometry (ICP-MS) 5 min, 15 min, 30 min, 45 min, 60 min, 4 h and 24 h after AuNPd injection. To prevent underestimation of gold concentration that could be due to an incomplete mineralization of nanoparticulate gold, solutions were dosed with calibration curves carried out with ionic gold standard solutions and gold nanoparticles standard solutions. No significant differences were noted whatever the standard solutions used, confirming validity of the measurement (data not shown). AuNPd blood concentration-time curve with a semilogarithmic scale evidenced a rapid drop in initial concentration, followed by more gradual decline fitting into a classical bi-compartmental pharmacokinetic model characterized by a fast initial distribution phase followed by a terminal elimination phase with slower rate (Fig. 3). Mean pharmacokinetic parameters, calculated using bi-compartmental analysis are summarized in Table 1. One hour after injection, more than 95% of the injected dose was eliminated from the blood circulation, which is consistent with the low value of T_1/2α_ (half-life for the distribution phase). T_1/2α_ is the time required for the blood level of AuNPd to decrease by 50% of its initial value. Gold was undetectable in blood 24 h post-administration, thus explaining the short T_1/2β_ (half-life for elimination) and the high clearance value. The V_d_ parameter that reflects the degree of how a foreign material is distributed in organs and tissues, was well below the volume of the mice body fluid (2 ml), which meant that AuNPd accumulated in the organs. All together, these data suggest that the amount of AuNPd in the central compartment (blood) declines rapidly due to the transfer of NP to the peripheral compartment (tissues) and/or to AuNPd elimination that occurs simultaneously.

**Table 1:** Pharmacokinetic parameters of AuNPd.

	AuNPd
T_1/2α_ (biodistribution half-life)	4.2 min
T_1/2ß_ (elimination half-life)	307.7 min
AUC (area under the curve)	4.7 µg.min.mL^−1^
Cmax	0.3 µg.mL^−1^
Cl (Clearance)	8.4 mL.min^−1^
Vd (Volume of distribution)	1.2 L

### Tissue biodistribution of AuNPd

**Figure 4:**
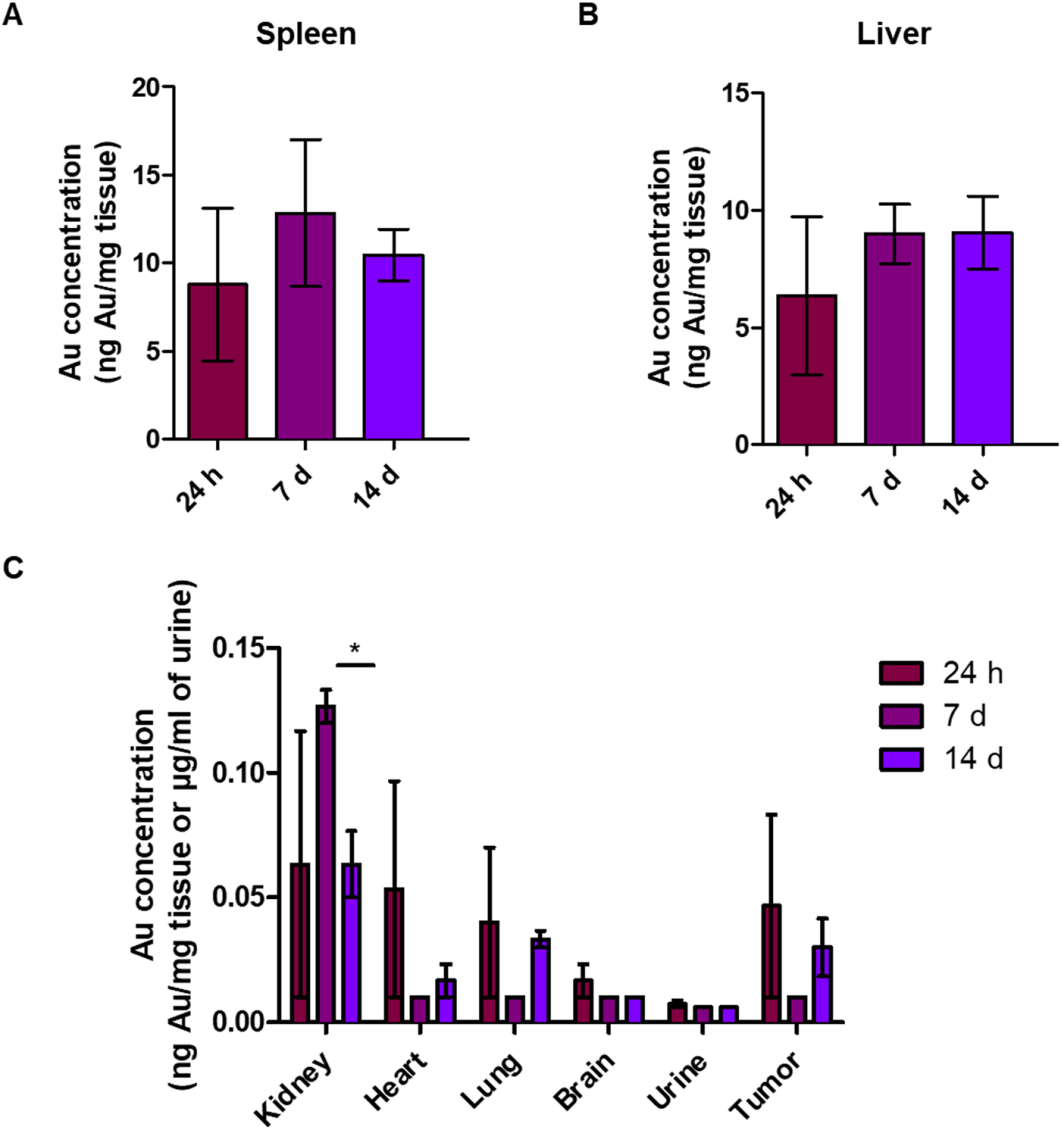
Gold concentration measured by ICP-MS 24 h, 7 days and 14 days after a single injection of AuNPd (1 mg/kg). (n = 3, data are mean ± SEM).

Pharmacokinetic parameters indicate a rapid clearance of AuNPd from the bloodstream. To determine the distribution of AuNPd in tissues, three groups of six mice were administered with 1 mg/kg of AuNPd and sacrificed at different times after injection: 24 h, 7 and 14 days. The control group was injected with vehicle alone and sacrificed at day 14. Gold concentration in various tissues, including liver, spleen, kidney, heart, lung and brain, was measured by ICP-MS and expressed as ng/mg organ. Twenty-four hours after injection, spleen and liver were preferential sites for gold accumulation with 8.78 ± 4.33 ng/mg and 6.36 ± 3.37 ng/mg (Fig. 4A et 4B), respectively. However, considering size of the organs, AuNPd accumulated mostly in liver, in which Au concentration was more than 35% of the injected dose after 24 h and more than 50% after one and two weeks (Table 2). In other organs and in tumor, gold concentrations were much lower with less than 0.15 ng/mg of tissue at 14 days. The lowest concentration was found in the brain with less than 0.03 ng/mg. Moreover, no statistical difference was found between gold concentrations at different time point in lungs, heart, brain and urine. In addition, AuNPd were not cleared by kidney because less than 0.1% of injected dose was found in urine (Fig. 4C). All together, our results indicate that AuNPd accumulated mostly in the liver, without obvious decrease for 14 days.

**Table 2:** Gold concentration expressed in percentage of injected dose in spleen, liver and kidneys.

Organ	Time post-injection	Au quantity per organ (% of injected dose)	
		Mean	SEM
Spleen	24 h	4.74	2.34
	7 days	6.93	2.25
	14 days	5.65	0.79
Liver	24 h	35.36	18.77
	7 days	50.07	7.05
	14 days	50.42	8.61
Kidney	24 h	0.12	0.10
	7 days	0.23	0.01
	14 days	0.12	0.02

### Histological analysis of tissues

**Figure 5:**
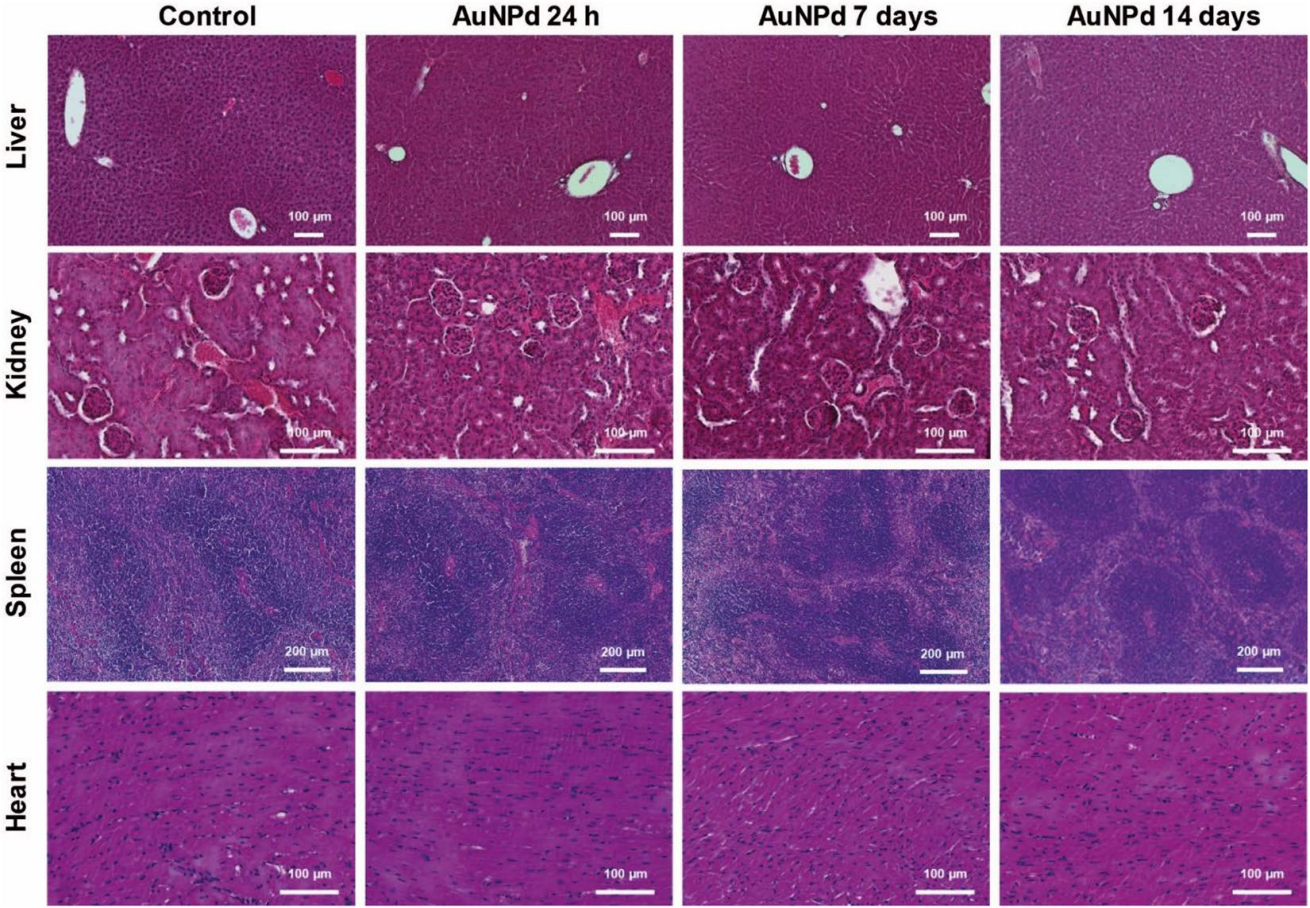
Histological section of mice liver, kidney, spleen and heart, 24 h, 7 and 14 days after intravenous administration of AuNPd (1 mg/kg) compared to the control group administered with vehicle alone. All sections were stained with hematoxylin-eosin.

Macroscopic examination of all organs including liver, spleen, kidney, heart, lung and brain tissue did not show any changes 24 h, 7, and 14 days after the administration of AuNPd (1 mg/kg), as compared to organs of control mice. No evidence of atrophy, hyperplasia, necrosis or inflammation was observed. To further explore for abnormalities, histological examination of tissues was performed using hematoxylin-eosin staining. Liver examination did not evidence any negative effects in animals injected with AuNPd compared to control group. The microscopic parenchymal architecture evidenced normal hepatocytes without any signs of steatosis, inflammation nor fibrosis. Similarly, microscopic observation of kidney, spleen and heart did not reveal any sign of inflammation or fibrosis (Fig. 5).

### Uptake and subcellular localization of AuNPd in hepatic and splenic tissues

**Figure 6:**
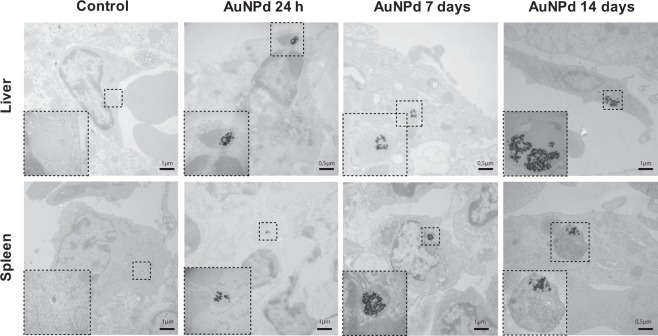
Representative TEM micrographs of Kupffer cells in liver and macrophages in spleen of mice 24 h, 7 and 14 days after intravenous injection of AuNPd (1 mg/kg) compared to the control group administered with vehicle alone. Magnified views of the square area show endolysosomal compartment containing clustered AuNPd in treated animals.

Biodistribution data have shown that AuNPd preferentially accumulated in liver and spleen. Therefore, we decided to assess subcellular localization of AuNPd in these organs by TEM at 24 h, 7 and 14 days after injection. A control group administered with vehicle alone was used. While gold is a dense-electron material, nanoparticles were easily detected on organ slides. AuNPd were observed in liver and spleen in all animals that receive AuNPd injection, and representative images are shown in Fig. 6. AuNPd were present inside phagocytosing cells of both liver (Kupffer cells) and spleen (macrophages). No AuNPd was detected in other liver cells like hepatocytes or endothelial cells nor in lymphocytes and red blood cells in spleen. Although some AuNPd were found isolated in Kuppfer cells and macrophages (data not shown), AuNPd preferentially agglomerated in cluster (containing more than 10 particles). Isolated AuNPd or AuNPd clusters were localized in endolysosomal compartments. No AuNPd were detected in cytosol, nucleus or in other cytoplasmic organelles. No differences in terms of AuNPd quantity and subcellular localization were observed between 24 h, 7 and 14 days post-injection. Kupffer cells and macrophages of control animals did not contain any AuNPd. In addition, no morphological changes such as chromatin condensation or mitochondrial morphology were detected in hepatic and splenic tissues of AuNPd injected animals compared to control mice. All together, our results show that AuNPd accumulate in endolysosomes without inducing cellular toxicity (Fig. 6).

### Biochemical and inflammation parameters after AuNPd injection

**Figure 7:**
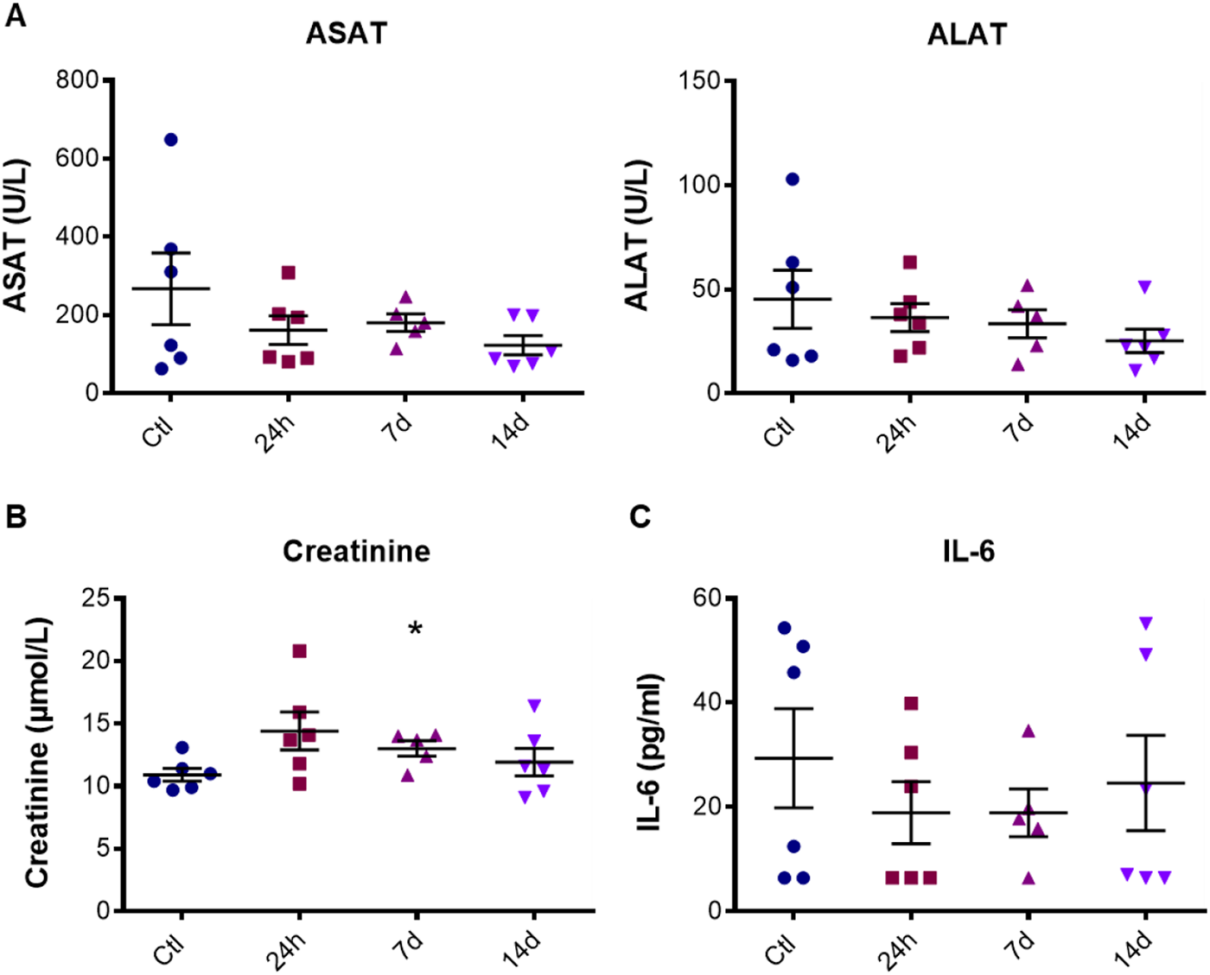
ALAT and ASAT values (**A**), creatinine level (**B**) and IL-6 concentration (**C**) in mouse plasma 24 h, 7 or 14 days after AuNPd injection (1 mg/kg) or 14 days after vehicle injection for the control group. Data represent the mean ± SEM of 6 mice and statistical significance was determined by Student’s t-test (*p < 0.05).

Finally, we investigated whether the treatment with AuNPd could modify the levels of biochemical tissue damage or inflammatory markers in plasma. To determine if AuNPd induced liver toxicity, we measured plasma ASAT (Aspartate Aminotransferase) and ALAT (Alanine Aminotransferase). An increase of these enzymes is considered as a key marker of hepatocellular injury. ALAT and ASAT levels remained normal in AuNPd treated animals for 14 days as compared to the control group with a mean value around 183 U/L and 35 U/L for ASAT and ALAT, respectively (Fig. 7A). These results, combined with histological analysis, indicate that even if AuNPd accumulate in liver, they do not alter hepatic functions. Kidney function was assessed by measuring creatinine plasma level. We did not observe any significant difference between the AuNPd treated groups sacrificed at 24 h and 14 days and the control group. However, the group sacrificed 7 days after AuNPd injection demonstrated a slight but statistically significant increase in creatinine level as compared to control group from 10.92 ± 0.51 to 13.02 ± 0.61 µmol/L (p = 0.026) (Fig. 7B). Importantly, these values remain under the toxicity threshold and are consistent with the literature (15–30 µmol/L)^55–57^. Moreover, the increase in creatinine was transitory and go back to normal value one week later, suggesting the absence of kidney dysfunction. Combined together, these results give evidence that AuNPd accumulation in liver and their small amount in kidney do not affect organ functionality.

The potential inflammatory effect of AuNPd was determined by plasma level of interleukin-6 (IL-6), a well-recognized mediator of inflammation. In control group, the average level of IL-6 was around 30 pg/ml as described in literature^56^. Comparing to the control group, IL-6 level was not significantly modified at 24 h, 7 days and 14 days post-treatment (Fig. 7C). These results confirmed that a unique intravenous injection of 1 mg/kg AuNPd was safe and did not induce acute and chronic toxicity.

## Discussion

This study focused on the *in vivo* pharmacokinetics, biodistribution and toxicity of AuNP synthetized by ablation laser in dextran solution, following a bolus intravenous administration to subcutaneous tumor grafted mice. The healthy behaviour of animals, as well as the absence of acute and chronic toxicity on kidney, spleen and liver, confirm the safety of AuNPd previously described *in vitro*^54^. Pharmacokinetic data have shown that AuNPd were promptly cleared from the bloodstream and accumulated preferentially in liver and spleen. Small quantities of gold were also present in kidney, lung, heart and brain. Our results are consistent with the pharmacokinetic profile described in the literature for AuNPd not coated with PEG^58^. Indeed, liver has largely been described as a key organ for nanoparticles accumulation (predominantly inside Kupffer cells) after the intravenous administration^59^. Form, size, charges and density of Au-NP modulate tissue distribution^60^. In a study on tissue distribution of spherical AuNPd in rat, Jong *et al*., show that large AuNPd (diameter between 50 and 200 nm) almost solely distributed in the blood, liver and spleen whereas smaller AuNPd (10 nm diameter) were largely distributed^61^. In our study, gold content in liver and spleen did not decreased over time, suggesting a poor clearing efficacy through the bile ducts. A very low amount of AuNPd (0.23% of the injected dose) was found in kidney 7 days after administration. The decrease in gold content in kidney measured at day 14 as compared with day 7 may be explained by the clearance in urine (≤0.03 ng/mL) of the smaller AuNPd (<8 nm) due to the physical size of glomerular filtration^62^. Because AuNPd predominantly accumulated in the liver, spleen and kidney, measurements of biochemical parameters and histopathological analysis were performed to deeply investigate their safety/toxicity risk for principal organs. Since plasma ALAT, ASAT activities and creatinine level remained almost unchanged and comparable to the parameters of the control mice group, one can conclude that the accumulation of AuNPd did not provoke any hepatic or renal toxicity. Furthermore, we detect no sign of histological damage such as fibrosis or inflammation in tissue. Lastly, the absence of chronic inflammation was supported by plasma IL-6 level that remains normal for 14 days after the AuNPd administration.

Since non-specific uptake of nanoparticles can occur in populations of phagocytic cells and dense capillary beds present in non-target organs, we also quantified gold in cardiac and pulmonary tissues. Very low concentrations of gold were observed in these organs, thus confirming a good tolerability of AuNPd. In accordance with previous data^26^, less than 1% of gold was detectable in brain. The smaller size of NP may have crossed the blood brain barrier (BBB) through the 20 nm space between astrocytic end-feet basement membrane and capillary endothelium^63^. Alternatively, adsorption of apolipoproteins on gold NPd observed in our previous study^54^ could have further facilitated brain transport through interaction of apolipoprotein with the scavenger receptor class B type located at the BBB^64^. Moreover, surfactants coated at the surface of nanoparticles may improve anticancer drug accumulation in brain tumors^65^. Importantly, no cognitive or compartmental abnormal sign was detected in living animals, suggesting that AuNPd can be useful for brain tumor treatment as drug delivery vector or photothermotherapy after optimizing brain uptake. Work is in progress in this direction to improve brain targeting by BBB opening using ultrasound. Focused ultrasound mediated-BBB opening is a non-invasive technique where ultrasounds are used to temporarily open the BBB in highly targeted areas of the brain^66^.

Accumulation of nanoparticles in tumor by a passive targeting way is in competition with the internalization and clearance from blood by phagocytic cells and macrophages^67^. In addition, nanoparticles are supposed to be small enough to reach the tumor through transvascular pores and fenestrations for efficient EPR effect. Here, gold was not detectable in tumor, which is consistent with the short blood half-life. Indeed, previous reports have shown that an increase in plasma half-life resulted in greater accumulation in the tumor^67^. However, the most efficient passive tumor targeting with AuNP remain lower than 8% of the injected dose per gram of tissue^68^. Moreover, Jinbin Liu *et al*., described a GS-AuNP with rapid clearance in normal tissue, long retention in tumors, and high tumor targeting specificity but less than 5% AuNP per gram of tissue in tumor was observed at 12 h post injection^69^. In order to increase AuNPd accumulation in tumor, the quantity of dextran at the surface of AuNPd should be increased or another surfactant such as polyethylene glycol (PEG) could be used. PEG has been previously shown to protect NP against opsonization and induce an increase in blood circulation time and enhanced tumor accumulation^70^. Alternatively, less negative zeta potential could increase the blood circulation time. Indeed, Arvizo *et al*. has found that neutral and zwitterionic AuNPd had a longer blood circulation time while negatively and positively charged AuNP had a relatively short blood half-lives^71^.

In general, the level of toxicity we observed appears to be very low compared to previous studies on the use of chemically-prepared NP^23–25^. It should be noted that the observed residual accumulation of gold in the organism was mentioned in almost all previous studies^23,24,71^. Nevertheless, our data show that the accumulation of laser-synthesized AuNP does not cause any acute or chronic toxicity, as it can take place with alternative Au-based counterparts or many other nanomaterials. As an example, residual accumulation of silica (SiO_2_) nanoparticles in liver under similar conditions induced mononuclear inflammatory infiltrate at the portal area and hepatocyte necrosis at the portal triads 7 days after injection^72^, as well the elevation in ASAT level and a larger number of sinusoidal Kupffer cells 48 h after intravenous administration of rats^55,72,73^. We suppose that Au nanoparticles could be completely eliminated from the organism if their size is within renal glomerular filtration range (<8 nm)^62^. It is important that laser-ablation technology makes possible the fabrication of both bare and polymer-coated AuNP within this range^46–48^. Interestingly, we recently carried our similar study of safety and biodistribution of laser-synthesized Si nanoparticles^56,74^, which present another promising material for imaging and therapy applications in biomedicine^75^, but in contrast to Au nanostructures^76^ Si nanoparticles are biodegradable^56,77,78^. Our experiments also evidenced preferable accumulation of bare (uncoated) Si NP in liver and spleen under their intravenous administration, but such an accumulation did not cause any toxicity effects^56,78^. In addition, in contrast to gold nanostructures Si NP were rapidly biodegraded in the organism and completely eliminated 4–6 days following their injection^56^.

## Conclusions

For the first time we studied safety, pharmacokinetics and biodistribution of laser-synthesized dextran-coated AuNP under intravenous administration in small animal model. We found that AuNP were mostly accumulated in liver and spleen, but did not result in any hepatic or renal toxicity, as was indicated by stability of biochemical parameters and histological examination of tissues. The safety of AuNPd was confirmed by healthy behavior of animals and the absence of acute and chronic toxicities in key organs. In general, despite residual accumulation in tissues, laser-synthesized nanoparticles present a safe and very promising object for biomedical applications and can be used us drug carriers, contrast agents or sensitizers of therapies.

## Methods

### Synthesis of Au nanoparticles

For the fabrication of dextran-coated Au nanoparticles, we used a Yb:KGW laser (Amplitude Systems, 1025 nm, 480 fs, 100–200 μJ per pulse, 1–100 kHz). The laser beam was focused onto the surface of s gold target (99.99%, GoodFellow, Lille, France) placed on the glass vessel bottom filled with 7 ml of deionized water containing 1 mg/mL of dextran (MW ~40,000, Sigma-Aldrich). To avoid the formation of craters leading to the decrease of ablation efficiency, the target was moved at a speed of 0.5 mm/s during the experiment. To measure concentration of formed nanoparticles, we measured weight of the ablated target before and after laser ablation process.

### Characterization of nanoparticles

We used a high-resolution transmission electron microscopy system (JEOL JEM 3010) to assess structural characteristics of formed nanoparticles. To prepare samples, we dropped a droplet (about 5 μL) of AuNP solution onto a carbon-coated TEM copper grid and dried it at ambient conditions. To examine extinction spectra, we used a UV–VIS spectrophotometer (UV–2600, Shimadzu).

### *In vivo* study design

All experimental protocols and animal analyses were conducted in accordance with the guidelines of the French Government and the Regional Committee for Ethics on Animal Experiments (authorization number 0100903). The experimental procedure was approved by the Committee for Ethics on Animal Experiments of the Institute of NeuroPhysiopathology. For the biodistribution study, 24 athymic nude female mice (Athymic Nude-Foxn1^nu^) (Harlan, France) aged 6 weeks were randomly divided in 4 groups. Mice were housed in cages, located in a well-ventilated, temperature-controlled room 21 ± 2 °C with relative humidity ranging from 40% to 60%, and a light/dark period of 12 h, with free access to water and food. On day 0, 2.5 × 10^6^ U87-MG human glioblastoma cells were administered subcutaneously on the left flank of all mice. Tumor growth and body weight were monitored twice a week. On day 14, when tumor measured approximately 100 mm^3^, 3 groups of 6 mice were intravenously administered in tail vein with a single dose of 1 mg/kg dextran-coated gold nanoparticles (AuNPd) diluted in phosphate buffer saline (PBS), corresponding to the maximal volume that can be administered intravenously. Control mice were injected through the tail vein only with PBS. After AuNPd administration, mice body weight and behaviour were monitored to detect a possible toxic effect of NPs. Animals were sacrificed at different times after AuNPd injection: 24 h, 7 days and 14 days. Twenty-four hours before sacrifice, three mice per group were housed individually in metabolic cages to recover urine. Mice were then anesthetized with a solution of ketamine (0.75 mg/kg body weight) and xylazine (0.10 mg/kg body weight), and exsanguinated by cannulating the posterior aorta. The liver, spleen, kidneys, lungs, heart, brain, tumor were removed and processed for histological and electron microscopy analysis as described below. Samples dedicated to gold determination were frozen and stored at −20 °C before analysis. The organs of 3 mice per group were used for gold determination and histological analysis. The organs of the 3 other mice were used for electron microscopy analysis.

For the pharmacokinetic study, 35 athymic nude female mice (Athymic Nude-Foxn1^nu^) (Harlan, France) aged 6 weeks were used and randomly divided in 7 groups. Mice were administered intravenously with the maximal dose of 1 mg/kg AuNPd. Animals were sacrificed at different time points: 5 min, 15 min, 30 min, 45 min, 60 min, 4 h and 24 h after AuNPd injection and blood samples (800 µL) were collected by intra-cardiac puncture. Samples dedicated to gold determination were frozen and stored at −20 °C before analysis.

### Gold determination content

Biological samples including liver, spleen, lung, kidney, heart, brain, tumor, were cut in small pieces and mineralized with nitric acid (3 M) / hydrochloric acid (1 M) and incubated at 100 °C during 8 h. Liquid samples including whole blood and urine were mineralized by addition of 1 ml of acid solution. Mineralized pellets were then diluted in deionized water and analyzed by ICP-MS using a Thermo Series II ICP-MS apparatus (Thermo-Electron, Les Ulis, France) to determine Au concentration. Standard calibration curve was performed with a solution of ionic gold and a solution of AuNP. (Quantification threshold was fixed at 0.01 ng/mg for tissues and 0.006 ng/µL for urine and blood).

### Biochemical analysis

Blood samples were collected by intra-cardiac puncture; plasmas were prepared by two successive centrifugations at 382 g (2000 rpm) for 20 minutes. Samples were stored at −20 °C until analysis by Institut clinique de la souris; Illkirch-Graffenstaden. ALAT (Alanine AminoTransferase), ASAT (Aspartate AminoTransferase) and creatinine plasmatic levels were quantified using AU400 Chemistry Analyzer, Beckman Coulter. Interleukin-6 plasmatic level was quantified by immunoassay using Mouse Cytokine/Chemokine Magnetic Bead Panel (IL-6) (Millipore, MCYTOMAG-70k).

### Histological analysis

Organs were carefully collected, fixed and conserved in formalin solution before paraffin-embedding. Three-µm-thick paraffin sections of different organs were then processed manually. Slides were deparaffinized in three successive baths of xylene (Hydroclear) for 10 min and then rehydrated twice with 100% ethanol. After rising in tap water for 10 min, slides were stained with Gründwald Hematoxylin for 30 s. After washing, slides were stained with eosin for 15 s and rapidly rinsed in tap water. Three baths of 100% ethanol were realized, and slides were stained in alcoholic safran solution for 30 seconds. Slides were then cleared in xylene three times and mounted with Eukitt medium. The stained slides were scanned on a whole slide scanner (Nanozoomer 2.0-HT, *Hamamatsu*, Japan) at × 40 magnification, obtained images were opened using NP-Viewer software.

### Transmission electron microscopy studies of biological samples

For TEM, samples were processed as followed. Three mice per group were perfused with 2.0% paraformaldehyde, 2.5% EM grade glutaraldehyde in PBS (pH 7.4). Organs were then carefully collected and cut into 1 mm^3^ cubes in perfusion solution under binocular loupe. Samples were then fixed in 2.0% paraformaldehyde, 2.5% EM grade glutaraldehyde, 0.1% tannic acid in 0.1 M sodium cacodylate buffer (pH 7.4). After fixation, samples were washed twice in 0.1 M sucrose 0. 1 M sodium cacodylate buffer (pH 7.4) and post-fixed in 2% osmium tetroxide in 0.1 M sodium cacodylate buffer (pH 7.4) for 1 h. Samples were washed once with 0.1 M sucrose 0.1 M sodium cacodylate buffer and thrice in deionized water to eliminate excess of osmium. Sample dehydration started with 15 min 30% ethanol bath and a second in 50% ethanol. At this step a first tissue contrast was realized in 1% uranyl acetate in 70% ethanol at 4 °C on night. Sample dehydration continued with 40 min successive baths of 70, 90, 95 and 100% ethanol and finished with a 100% acetone bath. Samples in solvent were progressively infiltrated with increasing concentration of Epon in an Epon/aceton mix. Once in 100% Epon, sample were disposed in Epon into flat embedding molds and put in oven at 60 °C for 48 h for polymerization. Ultrathin sections of 80 nm were cut with a diatom diamond knife on a LBK ultramicrotome Leica UCT and placed on coated square mesh copper grids. Sections were then counterstained with uranyl acetate and Reynold’s lead citrate and viewed with a FEI Tecnai G2 electron microscope. Digital images were acquired with a Veleta Camera (Olympus).

### Statistics

Data were analyzed for statistical significance using GraphPad Prism software.
